# There is no room for homelessness

**DOI:** 10.1016/j.lana.2023.100621

**Published:** 2023-10-20

Life, food, freedom, education, and healthcare are among the most fundamental and universal human rights. The constitutions of Guatemala, Iceland, Bangladesh, Nigeria, and Fiji, for example, all acknowledge at least some of the human rights of their citizens. All these countries protect the right to general education. Except for Iceland, these constitutions also recognise the right to safety of their citizens. However, only Bangladesh and Fiji have constitutions that specifically recognise the right to home or shelter. Is having a home a universal human right that should also be acknowledged in the constitutions of countries, like education? Is a home to stay and sleep in not an extra layer of security? Is having privacy in our own home not one of the most essential aspects of human dignity?

Constitutions have helped to provide a vital framework of hope and civilization to nations by regulating governments to set fundamental principles, rights, and obligations to people. The UN acknowledges that housing is a human right, as stated in the Universal Declaration of Human Rights (UDHR), signed in 1948. Perhaps the fact that most constitutions do not mention housing as a human right explains why governments do not see the problem of homelessness as a priority.

Although the definition of homelessness may appear straightforward (the condition of not having a home), it is actually complex. Here, we will use a general definition of homeless provided by The Substance Abuse and Mental Health Services Administration, a branch of the US Department of Health and Human Services: “an individual or family who lacks a fixed, regular, and adequate nighttime residence, such as those living in emergency shelters, transitional housing, or places not meant for habitation”. Thus, the quality of the house also matters. In fact, the UDHR also advocates for a standard of living (including housing) with dignity, that can promote health. Sadly, thousands of people in low-income and middle-income countries living in extreme poverty own precarious houses with roofs and walls made of cardboard or cane mats with no insulation, no basic services, and dirt floors. This situation does not seem to be much better in the poorest areas of the USA.

It is estimated that nearly 600,000 people in the USA are experiencing homelessness, that is about 0.18% of the population, most of whom are concentrated in the four most populated states: California, New York, Florida, and Texas. Approximately 70% of those who experience sheltered homelessness are men, and nearly 25% are under age 18. The situation in Latin America and the Caribbean (LAC) is also very alarming. Nicaragua (78%), Bolivia (75%), and Peru (72%) have the highest rates of families experiencing homelessness or those who live in poor-quality housing, whereas Costa Rica has the lowest rate (18%). In 2020, more than 200,000 Brazilians were homeless. The number of people experiencing homelessness in the USA and Brazil, and the high rates of people who are homeless without shelter or living in poor conditions in other countries should prompt governments to better tackle this problem, more rapidly and more effectively.

Several factors are associated with homelessness. A study conducted in California, the state with the highest number of people living without a roof in the USA, suggests that housing price is directly associated with homelessness. Loss of income is also a major driver, often associated with health crises. Another factor is the insufficient support that people experiencing homelessness receive from governments or charity agencies, sometimes due to a lack of awareness of the limited assistance programmes. Violence was noted as another reason among 15% of those experiencing homelessness. The factors associated with homelessness in LAC appear to be similar: high housing prices and land prices, low income, and loss of job. In Latin America, the paucity of proper urban planning seems to contribute to the sizeable proportion of improvised and unsafe houses.

Homelessness affects dignity and is associated with physical and mental health issues, drug addiction, alcoholism, and discrimination. Some recommendations have been suggested to tackle and prevent homelessness, including affordable housing, better salaries, financial support, and better medical care support and access (including for mental health), and orientation of alternative assistance programmes. LAC needs more affordable lands as people build their own houses at the pace they can afford. Natural disasters are also a cause of homelessness that governments should consider in their prevention policies. Naturally, the implementation of these recommendations requires a profound change in the economic plans of governments. However, this does not seem to be impossible. For example, Massachusetts has an emergency housing assistance programme, offering shelters to families with children and pregnant women with no other children. However, similar initiatives are needed across the USA and in other countries. Brazil has a subsiding programme for affordable housing, “*Minha casa, Minha vida*” (“my house, my life”), that has helped thousands of people since its start in 2009. This project now aims to build and deliver two million new houses by 2026.

The actual burden of homelessness in LAC has been little studied. Some reports are outdated and data for some countries are not available, making it even more difficult for governments to respond to this crisis adequately. However, although the crisis of homelessness in the USA is better documented, the number of people living in homeless conditions continues to increase. More research is urgently needed to capture the actual burden of homelessness in other regions and help policy makers to implement effective programmes. However, given the growing problem, there is no room for inaction from local, regional, and federal governments. On December 10, 2023, the UN will commemorate the 75th anniversary of the Human Rights initiative, and will remind us that housing is also a human right.

